# Prognostic Value of Immune-Inflammatory Index in PSI IV-V Patients with COVID-19

**DOI:** 10.1155/2021/9987931

**Published:** 2021-08-16

**Authors:** Rui Han, Honghui Su, Gangwen Guo, Qiao Wang, Jiahui Ma, Zhenxing Li, Shitong Huang, Yuncheng Ni, Rong Hu, Dong Huang, Haocheng Zhou

**Affiliations:** ^1^Department of Anesthesiology and Pain Medicine, The Third Xiangya Hospital, Central South University, Changsha, 410013 Hunan Province, China; ^2^Zhongfa Xincheng Branch of Tongji Hospital, Huazhong University of Science and Technology, Wuhan, Hubei Province, China

## Abstract

**Objective:**

Respiratory failure is the leading cause of mortality in COVID-19 patients, characterized by a generalized disbalance of inflammation. The aim of this study was to investigate the relationship between immune-inflammatory index and mortality in PSI IV-V patients with COVID-19.

**Methods:**

We retrospectively reviewed the medical records of COVID-19 patients from Feb. to Apr. 2020 in the Zhongfa Xincheng Branch of Tongji Hospital, Wuhan, China. Patients who presented high severity of COVID-19-related pneumonia were enrolled for further analysis according to the Pneumonia Severity Index (PSI) tool.

**Results:**

A total of 101 patients diagnosed with COVID-19 were identified at initial research. The survival analysis revealed that mortality of the PSI IV-V cohort was significantly higher than the PSI I-III group (*p* = 0.0003). The overall mortality in PSI IV-V patients was 32.1% (9/28). The fatal cases of the PSI IV-V group had a higher level of procalcitonin (*p* = 0.022) and neutrophil-to-lymphocyte ratio (*p* = 0.033) compared with the survivors. Procalcitonin was the most sensitive predictor of mortality for the severe COVID-19 population with area under receiver operating characteristic curve of 0.78, higher than the neutrophil-to-lymphocyte ratio (0.75) and total lymphocyte (0.68) and neutrophil (0.67) counts.

**Conclusion:**

Procalcitonin and neutrophil-to-lymphocyte ratio may potentially be effective predictors for mortality in PSI IV-V patients with COVID-19. Increased procalcitonin and neutrophil-to-lymphocyte ratio were associated with greater risk of mortality.

## 1. Introduction

The rapid spread of the Coronavirus Disease 2019 (COVID-19) epidemic has affected almost 190 million people across the world since December 2019. Respiratory failure is the leading cause of mortality in COVID-19, accounting for more than 56% of mortalities in hospitalized patients [1]. The risk factors of mortality include obesity, smoking, comorbid condition, and advancing age [2–5]. Early recognition of a severe case is essential to improve the clinical outcome and reduce mortality [6].

Pneumonia Severity Index (PSI) is a well-established severity assessment tool, which has been widely used to evaluate the severity of pneumonia caused by a bacterial or viral pathogen [7, 8]. The PSI scoring tool is based on 20 demographic, comorbid, and clinical variables. Higher PSI scores indicate greater risk of adverse outcome, such as ICU admission, mechanical ventilation, and mortality. The overall mortality rate ranged from 9 to 27% in severe pneumonia patients (defined as PSI class IV-V) [9]. Recently, PSI has been applied in severity assessment for COVID-19 patients. PSI could provide moderate to good predictive value in the mortality rate of COVID-19 [10, 11]. However, many fatal cases are asymptomatic or present mild symptoms after viral infection [4]. Thus, the criteria of real “severe cases” classified by the PSI system needs to be reconsidered.

One key feature of a fatal COVID-19 case is the generalized disbalance of inflammation. The latest systematical review has suggested the prognostic value of immune-inflammatory parameters in the progression of COVID-19 [12]. Specifically, severe COVID-19 patients are likely to present elevated levels of white blood cell (WBC), neutrophil counts, neutrophil-to-lymphocyte ratio (NLR), D-dimer, procalcitonin (PCT), C-reaction protein (CRP), and erythrocyte sedimentation rate (ESR) [11, 13–18]. On the other hand, the total lymphocyte count is decreased in severe cases [19, 20]. However, few studies have focused on the potential role of immune-inflammatory index in severe pneumonia patients with COVID-19. In this study, we analyzed the relationship between immune-inflammatory parameter and mortality in PSI IV-V patients with COVID-19.

## 2. Patients and Methods

### 2.1. Study Design

We conducted a retrospective review of all adult patients admitted to one temporary ward in the Zhongfa Xincheng Branch of Tongji Hospital, temporarily built for the management of COVID-19 patients between Feb. and Apr. 2020. The study was conducted in accordance with the Declaration of Helsinki and approved by the Third Xiangya Hospital. Due to the retrospective nature of the study, consent could be waived. The study was performed by one of the medical assistance teams from the Third Xiangya Hospital, Hunan, Changsha. This medical term was responsible for 54 beds in the ward. Severe and critically ill patients were transferred to one ICU with 30 beds if needed.

### 2.2. Patient Selection

All the enrolled patients had clear epidemic history, who were from the Wuhan district in the last 14 days before admission. In addition to contact history, COVID-19 patients either presented abnormal pulmonary CT imaging decribed as previously [21] or showed a positive PCR test for COVID-19.

### 2.3. Data Collection

One standard data collection form was used to collect the information of enrolled patients. with respect to the demographic, clinical, laboratory, and radiographic data. The first data available following admission was recorded and applied for further analysis. Missing data was assumed to be at normal range based on the regular principle of practice. Peripheral blood was obtained for the test of white blood cell (WBC), neutrophil, and lymphocyte counts; neutrophil-to-lymphocyte ratio (NLR), C-reactive protein (CRP), and procalcitonin.

### 2.4. Calculation of Pneumonia Severity Index

The Pneumonia Severity Index (PSI), also known as the PORT score was initially developed by Fine et al. to evaluate the severity of disease [9]. The calculation of PSI scores is based on 20 demographic, comorbid, and clinical variables. Patients were stratified into five risk categories according to the PSI scores. Patients with PSI class IV-V were considered as severe pneumonia, who should be admitted for further treatment.

### 2.5. Statistical Analysis

The Student *t*-test, Mann–Whitney *U* test, Chi-squared test, and the Fisher exact test were applied when appropriate. The Kaplan–Meier methods and log rank tests were used to determine survival curves for PSI I-III and IV-V patients. The receiver operator characteristic (ROC) curve was plotted using the variables of each immune-inflammatory index and the area under the curve (AUC) was calculated. Statistical significance was set at *p* < 0.05. Data were analyzed using Prism v8 (GraphPad, San Diego, CA, USA). Logistic analysis was performed by MATLAB vR2018b (MathWorks, Natick, MA, USA).

## 3. Results

### 3.1. Correlation between the PSI Scores and COVID-19 Severity

**Figure 1:**
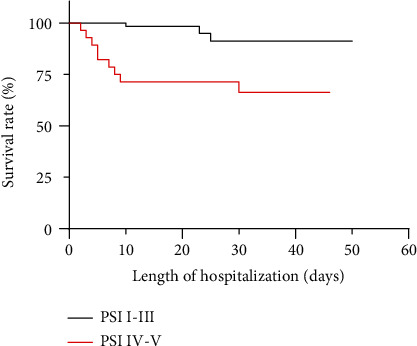
Survival curve according to the PSI scores.

**Figure 2:**
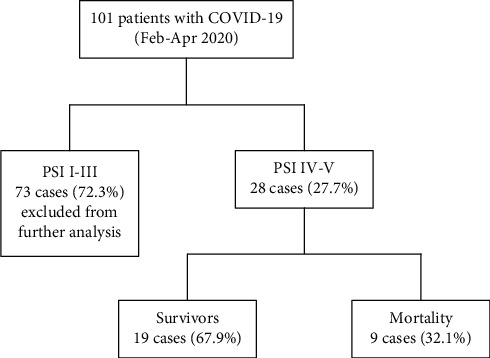
Flow chart of patients with COVID-19, scored by the PSI tool.

101 patients diagnosed with COVID-19 were confirmed at initial search. The COVID-19 patients were classified into two groups according to the PSI scores. That is, 72.3% of the patients (73 of 101) presented mild symptoms (PSI I-III) at admission. 28 (27.7%) patients were classified into PSI IV-V (Figure 1). Next, we compared the survival curves for each group (Figure 2). The findings showed that those with higher PSI scores had a greater mortality and shorter survival duration than the mild symptom cohort (*p* = 0.0003). Specifically, most PSI IV-V patients died within 10 days after admission. The majority of fatal cases occurred within 30 days.

### 3.2. Demographics of the PSI IV-V Patients

The PSI IV-V cohort was divided into two subgroups based on the clinical outcome (Table 1). Nineteen (67.9%) patients survived, and nine (32.1%) passed away. The average age was 73.4 ± 10.3 and 62.6 ± 9.0 years for the survivor and mortality groups, respectively. Hypertension was the most common comorbidity in all enrolled patients (*n* = 20/28). The incidence of hypoxemia (PaO_2_ < 60 mm Hg) was significantly associated with fatal outcome (*p* = 0.05). As for the immune-inflammatory parameter, the median concentration of procalcitonin was 0.12, 0.31 for the survivor and nonsurvivor groups. The risk of adverse outcome also increased with higher neutrophil-to-lymphocyte ratio, as shown in Table 1. However, the difference in total white blood cell, neutrophil, and lymphocyte counts and C-reaction protein was not significant in this study.

### 3.3. Performance of Immune-Inflammatory Index in Prediction for Mortality

The predictive performance of each immune-inflammatory parameter at different cut-off values for COVID-19 mortality is given in Table 2. PCT and neutrophil counts gave similar predictive sensitivity for COVID-19 mortality at all cut-offs. In addition, PCT provided the highest NPV in fatality prediction (100%, 86.7%, and 77.3%).

**Figure 3:**
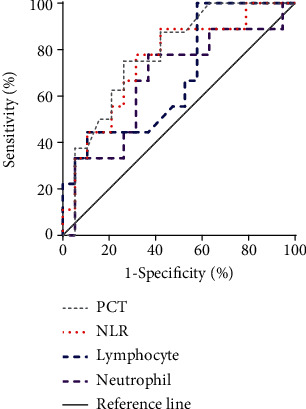
Receiver-operating characteristic curves of each immune-inflammatory index.

The ROC curves for prediction of COVID-19 mortality for each immune-inflammatory parameter are shown in Table 2 and Figure 3. The area under ROC curve of PCT was 0.78 (95% CI: 0.60 to 0.96), which was higher than the NLR (AUC: 0.75; 95% CI: 0.56 to 0.95), neutrophil (AUC: 0.67; 95% CI: 0.44 to 0.89), and lymphocyte counts (AUC: 0.68; 95% CI: 0.56 to 0.95).

## 4. Discussion

Respiratory failure is the most common and fatal complication in severe pneumonia patients with COVID-19. One key feature of the severe cases is the generalized disorder of the immune system, which subsequently contributes to the disease progression. Thus, it is essential to determine the relationship between dysfunctional immune responses and COVID-19 mortality, especially for those with severe pneumonia.

To elucidate the risk factor for COVID-19 mortality, we retrospectively reviewed the medical records of 101 patients. By introducing the well-established severity assessment tool (Pneumonia Severity Index (PSI)), patients were classified into two subgroups. Patients who presented with mild-moderate pneumonia were scored with PSI class I to III, and severe cases were scored with PSI class IV to V. Consistent with a previous study [9], the risk of COVID-19 mortality increased significantly with higher PSI scores. The general mortality ratio of COVID-19 patients was 2.7% (2 out of 73) for the nonsevere group and 32.1% (9 out of 28) for the severe cohort. Our finding suggests that COVID-19 patients presented greater risk of adverse clinical outcome compared with other typical community-acquired pneumonia [22, 23]. Likewise, recent studies [10, 11] have demonstrated moderate to good predictive performance of the PSI tool, in prediction for clinical outcome among the COVID-19 population. However, the validation of additional index such as erythrocyte sedimentation rate added into the PSI tool remains controversial [11].

The potential diversity of immune responses to SARS-CoV-2 is associated with the disease severity [24]. The characterized feature of immune dysfunction includes increased white blood cell and neutrophil counts, neutrophil-to-lymphocyte ratio (NLR), procalcitonin (PCT), C-reaction protein, erythrocyte sedimentation rate (ESR), and reduction of total lymphocyte counts [12]. However, previous data mainly focus on the general infected population with mild to severe symptoms. The prognostic value of these immune-inflammatory parameter in severe COVID-19 patients remains uncertain. In this study, we found that the difference of WBC and neutrophil counts and CRP is not significant in severe cases with COVID-19. Only PCT and NLR are sensitive predictors for mortality. Given that only severe pneumonia cases were included, the optimal cut-off threshold of NLR (8.3) for mortality was much higher than the value (NLR < 3.13) reported previously [14]. Thus, the higher levels of PCT and NLR may be effective predictors of COVID-19 death, especially for the severe pneumonia cases. In addition to COVID-19, accruing evidence has demonstrated that NLR can be a useful biomarker of cerebral infarct [25], cerebral hemorrhage [26, 27], major cardiac events [28], cancers [29], sepsis, and other infectious pathologies [30]. The easy and well-established test may help physicians improve the clinical decision in time.

Our study has several limitations. First, the number of participants was relatively small. We think it necessary to examine the validation of immune-inflammatory parameters in a larger cohort, especially for the severe cases. Second, some important immune-inflammatory parameters, such as interleukin 6 and tumor necrosis factor- (TNF-) *α*, were not evaluated due to the retrospective study design. Finally, we only observed the clinical outcome during hospitalization; the long-term outcome may be useful for the decision of public health strategy in the future.

## 5. Conclusion

Taken together, our findings suggest that procalcitonin and neutrophil-to-lymphocyte ratio may potentially be an effective predictor for mortality in PSI IV-V patients with COVID-19. Increased levels of procalcitonin and neutrophil-to-lymphocyte ratio were associated with greater risk of mortality.

## Figures and Tables

**Table 1:** Demographic data, comorbidities, and clinical characteristics of patients (*n* = 28).

Variables	PSI IV-V		*p* value
	Survivors (*n* = 19)	Nonsurvivors (*n* = 9)	
General demographics			
Age (years)	73.4 ± 10.3	62.6 ± 9.0	0.013
Sex (female), *n* (%)	8 (42)	3 (33)	>0.999
Comorbidities, *n* (%)			
Hypertension	12 (63)	8 (89)	0.214
Diabetes mellitus	5 (26)	4 (44)	0.407
Neoplasm	5 (26)	0 (0)	0.144
Chronic lung disease	9 (47)	1 (11)	0.098
Chronic liver disease	1 (5)	0 (0)	>0.999
Chronic renal disease	2 (11)	2 (22)	0.558
Smoker	4 (21)	2 (22)	>0.999
Alcohol abuser	0 (0)	0 (0)	>0.999
Laboratory			
PSI-related variables			
pH	7.5 ± 0.1	7.4 ± 0.1	0.281
BUN (mmol/L)	6.1 ± 2.8	8.0 ± 3.6	0.146
Sodium (mmol/L)	137.9 ± 5.2	138.0 ± 6.3	0.978
Glucose (mg/dL)	130.8 ± 51.1	182.6 ± 82.2	0.050
Hct (%)	35.6 ± 5.2	35.1 ± 5.6	0.795
PaO_2_ (mmHg)	65.4 ± 10.5	57.7 ± 12.6	0.133
Immune-inflammatory index			
WBC (×10^9^/L)	8.0 ± 3.0	9.4 ± 3.5	0.439
Neutrophil (×10^9^/L)	6.5 ± 2.9	9.2 ± 2.7	0.172
Lymphocyte (×10^9^/L)	0.9 ± 0.6	0.5 ± 0.3	0.129
NLR	10.8 ± 9.1	21.6 ± 14.6	**0.033**
CRP (mg/L)	65.4 ± 55.9	86.6 ± 49.8	0.232
Procalcitonin (ng/mL)	0.2 ± 0.3	0.4 ± 0.2	**0.022**
ICU admission, *n* (%)	2 (11)	6 (67)	0.005

BUN: blood urea nitrogen; CRP: C-reactive protein; Hct: hematocrit; ICU: intensive care unit; NLR: neutrophil-to-lymphocyte ratio; WBC: white blood cell.

**Table 2:** Sensitivity, specificity, positive predictive value, and negative predictive value of each immune-inflammatory index parameter for COVID-19 mortality prediction.

Immune-inflammatory index parameter	Sensitivity (95% CI)	Specificity (95% CI)	PPV (95% CI)	NPV (95% CI)	Area under the ROC curve (95% CI)
Neutrophils (×10^9^/L)					0.67 (0.44–0.89)
1st tertile	100 (67.6–100)	31.6 (15.4–54.0)	38.1 (20.8–59.1)	100 (70.0–100)	
2nd tertile	87.5 (52.9–99.4)	63.2 (41.0–80.8)	50.0 (26.8–73.2)	92.3 (66.7–99.6)	
3rd tertile	37.5 (13.7–69.4)	79.0 (56.7–91.5)	42.9 (15.8–75.0)	75.0 (53.1–88.8)	
Lymphocytes (×10^9^/L)					0.68 (0.47–0.90)
1st tertile	55.6 (26.7–81.1)	15.8 (5.5–37.6)	23.8 (10.6–45.1)	42.9 (15.8–75.0)	
2nd tertile	44.4 (18.9–73.3)	47.4 (27.3–68.3)	28.6 (11.7–54.7)	64.3 (38.8–83.7)	
3rd tertile	0 (0–29.9)	68.4 (46.0–84.6)	0 (0–39.0)	59.1 (38.7–76.7)	
NLR					0.75 (0.56–0.95)
1st tertile	88.9 (56.5–99.4)	31.6 (15.4–54.0)	38.1 (20.8–59.1)	85.7 (48.7–99.3)	
2nd tertile	77.8 (45.3–96.1)	63.2 (41.0–80.9)	50.0 (26.8–73.2)	85.7 (60.1–97.5)	
3rd tertile	44.4 (18.9–73.3)	84.2 (62.4–94.5)	57.1 (25.1–84.2)	76.2 (54.9–89.4)	
PCT (ng/mL)					0.78 (0.60–0.96)
1st tertile	100 (67.6–100)	36.8 (19.2–59.0)	40.0 (21.9–61.3)	100 (64.6–100)	
2nd tertile	75.0 (40.9–95.6)	68.4 (46.0–84.6)	50.0 (25.4–74.6)	86.7 (62.1–97.6)	
3rd tertile	37.5 (13.7–69.4)	89.5 (68.6–98.1)	60.0 (23.1–92.9)	77.3 (56.6–89.9)	

CI: confidence interval; NLR: neutrophil-to-lymphocyte ratio; NPV: negative predictive value; PCT: procalcitonin; PPV: positive predictive value; ROC: receiver operator characteristic.

## Data Availability

The authors declare that all the data supporting the findings of this study are available from the corresponding author upon reasonable request.
